# The development of vestibular system and related functions in mammals: impact of gravity

**DOI:** 10.3389/fnint.2014.00011

**Published:** 2014-02-07

**Authors:** Marc Jamon

**Affiliations:** Faculté de Médecine de la Timone, Institut National de la Santé et de la Recherche Médicale U 1106, Aix-Marseille UniversityMarseille, France

**Keywords:** vestibular development, critical period, otolith, utricle, microgravity, hypergravity, ontogeny, altricial

## Abstract

This chapter reviews the knowledge about the adaptation to Earth gravity during the development of mammals. The impact of early exposure to altered gravity is evaluated at the level of the functions related to the vestibular system, including postural control, homeostatic regulation, and spatial memory. The hypothesis of critical periods in the adaptation to gravity is discussed. Demonstrating a critical period requires removing the gravity stimulus during delimited time windows, what is impossible to do on Earth surface. The surgical destruction of the vestibular apparatus, and the use of mice strains with defective graviceptors have provided useful information on the consequences of missing gravity perception, and the possible compensatory mechanisms, but transitory suppression of the stimulus can only be operated during spatial flight. The rare studies on rat pups housed on board of space shuttle significantly contributed to this problem, but the use of hypergravity environment, produced by means of chronic centrifugation, is the only available tool when repeated experiments must be carried out on Earth. Even though hypergravity is sometimes considered as a mirror situation to microgravity, the two situations cannot be confused because a gravitational force is still present. The theoretical considerations that validate the paradigm of hypergravity to evaluate critical periods are discussed. The question of adaption of graviceptor is questioned from an evolutionary point of view. It is possible that graviception is hardwired, because life on Earth has evolved under the constant pressure of gravity. The rapid acquisition of motor programming by precocial mammals in minutes after birth is consistent with this hypothesis, but the slow development of motor skills in altricial species and the plasticity of vestibular perception in adults suggest that gravity experience is required for the tuning of graviceptors. The possible reasons for this dichotomy are discussed.

## Introduction

Gravity has modeled the evolution of life on Earth, and provides the frame of reference for the body orientation and the integration of accelerations in the various planes of space. Given the importance, ubiquity and stability of the gravitational force during the evolution of life, the organisms have the opportunity to develop without the need to adjust their gravity sensing to the external environment. It seems nevertheless that, in addition to a genetically controlled phase of development for target finding, a stimulus controlled phase is required for the fine tuning of synaptic terminals (Bruce, 2003). Studying the consequences of the development in altered gravity is of prime importance to understand how the system proceeds, and to envisage the consequences for long term space conquest.

The detection of the gravitational force requires specific receptors in charge of detecting linear accelerations. Two otolithic organs of the vestibular system, the utricle and saccule, achieve this function. These gravity receptors utilize a layer of otoconia, consisting of a complex arrangement of mineral and organic substance, that lies over sensory receptor areas. The shearing force produced by the inertial mass of otoconia displaced against the stereocilia of the sensory hair cells allows the detection of linear accelerations, and gravity. In mammals, as in reptiles and birds, the otoconia exhibit the crystallographic structure of calcite (calcium carbonate). On the sensory epithelium, two types of hair cells detect the movement of the otoconia layer: the flask-shaped type I cells, surrounded by an afferent nerve calyx, and the cylindral-shaped type II cells, contacted by afferent buttons. Bipolar vestibular neurons localized in Scarpa's ganglia connect monosynaptically the hair cells and reach the second order vestibular neurons localized in brainstem vestibular nuclei. The vestibular nuclei receive also projections from other sensory modalities including proprioceptive afferences originating mainly from the cerebellum. The otolithic information is thus integrated with the vestibular information from the semi-circular canals, and with other sensory systems such as vision and proprioception. It participates to various functions by means of afferent fibers sent to different organs through vestibular pathways that project to a variety of brain targets.

A basic function of the vestibular system is to maintain the body equilibrium in the gravitational field. This function requires a permanent control of the head and trunk position in space, and a control of the head in relation to the trunk. Gaze and postural stabilization result from a complex multisensory integration. The vestibulo-ocular tracts are involved in the movement of eyes to maintain the gaze, and the vestibulo-colic tracts innervate the neck muscles to support the head. The vestibulo-spinal tracts innervate the motor neurons of proximal and axial muscles of upper and lower extremities to maintain posture and balance.

The vestibular system has also privileged relationships with the cerebellum through vestibulo-cerebellar and cerebello-vestibular pathways. The gravisensing otolithic organs make direct and indirect connections with several sub regions of the cerebellum, particularly the floculo-nodular lobe, that constitutes the vestibular cerebellum. The cerebellum is a structure critical for the motor control coordination, the timing of movement but is also involved in motor learning and cognition (Fiez, 1996; Ito, 2006).

In addition to the role of the vestibular system in perception, oculo-motor and postural control, there is an increasing evidence for an important role in maintaining and organizing the navigation maps. The internal representation of head and trunk movements processed by the vestibular nuclei influence various cortical areas at the origin of the perception of egomotion. The second order neurons located in the vestibular nuclei project to thalamic nuclei where they converge with visual and somatosensory tracts (Shiroyama et al., 1999). Three main vestibulo-thalamic pathways are involved in the vestibulo-somatosensory and motor functions, in the vestibulo-striatal motor functions, and the vestibulo-visual and visuo-motor functions, respectively. The thalamic neurons process and relay information to various cortical areas (Lopez and Blanke, 2011) including the vestibular somatosensory cortex, the primary and premotor cortex, the cingulate cortex and the hippocampus, where the head direction cells and place cells seem to be strongly tuned to vestibular input. Head direction cell signal is a representation of an animal's perceived directional heading with respect to its environment. This signal appears to originate in the vestibular system (Taube, 2007). Current models suggest that otolithic information is involved in the perception of directional heading (Yoder and Taube, 2009). This function requires also the vestibulo-cerebellar pathway (Rochefort et al., 2013).

They are also accumulated evidence of the involvement of vestibular system in regulating the autonomic system. The stimulation of vestibular fibers modulates the activity of sympathetic fibers. For instance the vestibular system is involved in the regulation of the arterial pressure (Kerman and Yates, 1999) and bone mineralization (Denise et al., 2009). The vestibular nuclei could also regulate autonomous functions through a vestibulo-hypothalamic linkage (Fuller et al., 2002; Murakami et al., 2002).

## Development of gravity sensing

The maturation of gravity sensing requires the development of various levels of integration whose basic structures are mainly genetically programmed, but may depend in part on the exposure to the gravitational stimuli (Fritzsch et al., 2001; Fritzsch, 2003). An expanding set of data shows that the development of sensory functions needs the assistance of environmental information, during a critical period of their development, as was shown for hearing (Tees, 1967), vision (Hubel and Wiesel, 1970), and touch (Simons and Land, 1987), and to some extent olfaction (Poo and Isaacson, 2007). As for the other sensory information, the nervous system probably needs environmental experience to calibrate the gravity information during critical periods of the development. This hypothesis was evoked many times (Walton et al., 1992; Ronca and Alberts, 2000; Wubbels et al., 2002) and is one of the key issues in the developmental biology research in space (Moody and Golden, 2000). The existence of a critical period in the development of the vestibular system was reported in fish developed in microgravity (Moorman et al., 2002) or hypergravity (Wiederhold et al., 2003b), as in amphibians (Horn, 2004), and could be a general rule in the development of the vestibular sensitivity. The delimitation of a critical period is complex because the adaptation to gravity involves many structures and functions which mature with a different time schedule, and the complete maturation of the vestibular sensitivity requires a long delay.

The development of vestibular organs follows about the same progression in rats and mice. It starts on the second gestational week and complete maturation is achieved by the 4th postnatal week. The development starts with the formation of the otic placode at E8 (E: embryonic day). At E11 the endolymphatic canal forms, and anterior and posterior semi-circular canals appear at E12. The utriculo-saccular canal and the ampullar crests of semi-circular canals are apparent at E15. The proliferation of hair cells precursors may be set aside as early as E10.5 (Fritzsch et al., 2002; Beisel et al., 2005). Hair cells of the utricular macula begin to divide between E14 and E18 with a gradient from the center to the periphery. They are capable of mechano-transduction from E16 (Geleoc and Holt, 2003). They differentiate in type I and II between E16 and E18 (Kawamata and Igarashi, 1993), and most hair cells are formed at birth. Meanwhile the otoconia form between E14 and E16 (Anniko, 1980) and have fully matured at birth. In parallel with the peripheral organ, the first order vestibular neurons of the vestibulo-cochlear nuclei develop between E11 and E18, and the second order vestibular nuclei differentiate between E12 and E14 (Maklad and Fritzsch, 2003a). The synaptic contacts with sensory epithelium develop between E18 and the end of first postnatal week (Mbiene et al., 1988). The type I cells are only partly surrounded by the calyces until birth. The first calyces with adult type appear at PND4 (PND: postnatal day), and the innervation is comparable to the adult at PND10 (Desmadryl and Sans, 1990).

At birth the vestibular structures are therefore morphologically well developed, but they continue to mature. The number of hair cells increases from PND0 to PND3 then decreases from PND3 to PND7, in relation with a process of apoptosis that started at E19 and reach a peak at PND3 to decrease at PND7 (Zheng and Gao, 1997). The cilia are well differentiated at PND7, and they reach their definitive length at PND32. The utricle and saccule continue to grow until PND32 (Dechesne et al., 1986). The neurons of the vestibulo-cochlear nuclei and the second order vestibular nuclei continue to mature during the two first postnatal weeks (Curthoys, 1979b; Desmadryl, 1991). The vestibular apparatus becomes mature at the end of first postnatal month.

The projections of saccular and posterior cristae are the first afferent fibers to penetrate the cerebellum at E17.5, they reach the uvula and nodulus (Maklad and Fritzsch, 2003b). The cerebellar anlage has occurred between E9.5 and E11.5 (Chizhikov and Millen, 2003). Purkinje cells are born around E13, at which time they migrate into the cerebellar anlage, and granule cells migrate at the same time (Wang and Zoghbi, 2001). At PND1 the internal granule cell layer becomes recognizable and most granule cells mature between PND4 and PND20, with a peak of synaptogenesis of unipolar brush and granule cells at PND 13. The formation of inner granule cell layer, and granule cells neurogenesis, correlate with an extensive penetration of primary vestibular afferent. Axonal branches of primary vestibular afferent spread into the cerebellar cortex around PND7. Supernumerary climbing cells are eliminated during a critical period lasting between PND15 and PND16 (Kakizawa et al., 2000). This time corresponds to the final maturation of the cerebellum. The state of monoinnervation is achieved at the end of third postnatal week.

The axons originating from the lateral vestibular nuclei reach the cervical cord at E13–E14, the thoracic level one day later, and the lumbar cord before birth. About 40% of the vestibulo-spinal axons are present in the lumbar cord at birth and the adult pattern is observed at the end of the second postnatal week (Vinay et al., 2000, 2005). The percentage of ankle extensor neurons recruited by ventral horn stimulation in isolated brainstem/spinal cord preparations increases from 3% at birth to 35% at PND3–5 (Brocard et al., 1999). Thus the influence of the pathways involved in innervating antigravity muscles increases during the first postnatal week. The arrival of serotoninergic projections to the lumbar cord is critical for the development of locomotion (Vinay et al., 2000).

At functional level, the first regular afferent discharges appear in the peripheral apparatus at PND4. They increase progressively until PND30 (Curthoys, 1979a; Desmadryl et al., 1986). Vestibular evoked potentials in responses to linear accelerations appear between PND6 and PND8 (Freeman et al., 1999). Nevertheless the immature neural substrate is clearly capable of transducing vestibular input (Krasnov, 1991). First order vestibular neurons respond to low frequency acceleration from birth. Not only the major milestones of vestibular morphological vestibular development occurs prenatally, the system is functioning before birth as well (Ronca et al., 2008).

## Effects of altered gravity on the development of gravity sensing

It is impossible to remove gravity on Earth, thus experiments of deprivation of gravitational stimulus during rat development were limited to the exposure to microgravity during space flight. The missions embarked either pregnant females (Cosmos 1514: E12–E17; STS 66: E8–E19; STS70: E10–E19) or nursing litters (STS-72: PND14–PND30; STS-90: PND8–PND24), but longer exposures were not available due to the technical limitations of shuttle flight. Information was more easily obtained from ground experiments using centrifuges to produce hypergravity. Nevertheless the interpretation of these experiments is limited by the different periods of exposure, the intensity of gravity, the type of centrifuge itself, the age of testing and the species used. The alteration of gravity environment during the development involved changes at various levels of integration of the signal, often with opposite effects between hypergravity and microgravity conditions.

### Otoconia

Several studies showed that the size of otoconia is regulated to achieve a desired weight in a graded manner when the animals are subjected to altered gravity during the development of their statholits. Consequently the size of otoconia is increased in microgravity, and decreased in hypergravity. This process was observed in different species including snails (Wiederhold et al., 2003a), aplysia (Pedrozo et al., 1996), fish (Wiederhold et al., 1997; Anken et al., 2001, 2002a,b; Wiederhold et al., 2003a,b), Xenopus (Lychakov and Lavrova, 1985), chicken (Hara et al., 1995), hamsters (Sondag et al., 1996), rats (Lim et al., 1974; Krasnov, 1991). At variance the otoconia of animals exposed to altered gravity at maturity did not show any change (Lim et al., 1974; Ross et al., 1985; Hara et al., 1995; Sondag et al., 1995). Some of these results supported the existence of a critical period during the development of otoliths (Wiederhold et al., 2003a,b).

### Sensory epithelium

The exposure to altered gravity also affects the sensory epithelium with variable consequences depending on the period of development. Rats centrifuged (1.75 and 2G) from E9 and sacrificed at E19 showed an increased innervation of vestibulo-cerebellar fibers in the utricle (Bruce et al., 2006). This result, in a period that corresponds to the connection of vestibular afferents to the calyces, suggested that the increased stimulation of hair cells increased the rate of maturation of the utricle. At variance a decreased field of innervation was observed in rats exposed to microgravity during the same period (Bruce and Fritzsch, 1997; Bruce, 2003; Bruce et al., 2006). On the other hand, rats centrifuged (2G) from E9 until 1 week after birth did not show any modification in the time course of the establishment of fibers and calyces (Gaboyard et al., 2003). Thus the peripheral neuritogenesis was not modified by hypergravity 1 week after birth. At variance the utricles showed a delayed terminal location of the microvesicles at the apex of calyces that corresponded rather to a delay in the maturation of type I hair cells. Hypergravity was estimated to delay the synaptic stabilization of hair cells by 4 days (Brugeaud et al., 2006). At variance, rats exposed to microgravity postnatally (from PND8 to PND25) did not show any defect in the pattern of development (Dememes et al., 2001). These results support the hypothesis of a critical period for vestibular plasticity to occur between birth and PND8.

Overall these results suggest that the overstimulation of hair cells during the prenatal period of the formation of the epithelia contributes to accelerate the development of the sensory connections, but after birth the system corrects the tuning to adapt to the over stimulation with durable modifications of the vestibular epithelia.

### Vestibular nuclei

Exposure to altered gravity during the vestibular development has also consequences on the vestibular nuclei. In fish, the exposure to microgravity increased the number of synapses in some vestibular nuclei (Anken et al., 2002c). In newborn rats the exposure to microgravity induced a delay in the maturation. Rats exposed to microgravity from half their gestational period showed a delayed synaptogenesis of vestibular nuclei (Savel'ev et al., 1998; Yates et al., 2003) and altered morphology of the cortex, cerebellum and vestibular system (Keefe et al., 1986) that were interpreted as signs of retarded cell development and migration (Alberts et al., 1985). Delayed neuronal development and cell migration was observed immediately after flight (Malacinski et al., 1989), but no change persisted in 15 days old rats. Rats flown prenatally from E8 to E19 showed decreased projections from the saccule to the medial vestibular nucleus, with a reduced branching of the axons, while other vestibular afferents, probably those transducing angular accelerations may have compensated synaptogenesis by increasing synapses number in vestibular nuclei (Ronca et al., 2008). The preponderance of canalar synapses was interpreted as the result of the over stimulation of angular accelerometers due to the abundant rolling movement of the dam during the space flight (Ronca et al., 2008). Even rats exposed to microgravity only during their postnatal development (from PND8 to PND24) showed markedly smaller vestibular cell bodies and less growth of dendritic cell branching with a lack of development, paucity of cerebellar projections to vestibular nuclei (Raymond et al., 2003), and a reduction in motor dendritic tree size and complexity (Walton et al., 2003). These results suggested that the period of sensitivity persisted during the second and third postnatal week in vestibular nuclei, at variance with the sensory epithelium. Contrary to the microgravity, rats centrifuged (1.5G) from E9 to E19 showed a complex segregation of terminal fields of saccular axons into the four laminae (Bruce and Fritzsch, 1997; Bruce, 2003). This result was opposed to the poorly developed saccular axons in the medial vestibular nuclei of rats exposed to microgravity during the same period. The faster rate of vestibular maturation of rats exposed to hypergravity was confirmed by the faster maturation of peripheral utricular connections in rats centrifuged (1.5 and 2G) from E10 to E20 (Bruce et al., 2006). These results suggested that the exposure of microgravity retarded the development while the exposure to hypergravity advanced the maturation.

### Cerebellum

Effects of microgravity exposure on the vestibular nuclei of rats flown between PND8 and PND24 showed that space flight profoundly affects the postnatal development of cerebellar branching in the vestibular nuclei of rats (Raymond et al., 2003). The effects of hypergravity on the vestibulo-cerebellar connections were analyzed in details by Sadjel-Skulkowska and collaborators. Their experiments on rats exposed to hypergravity (1.5 and 1.75G) during the almost complete development of vestibular system (from E8 to PND20) showed a decreased mass of the forebrain and particularly of the cerebellum at PND6-PND9 then at PND21 (Sajdel-Sulkowska et al., 2001; Ladd et al., 2006), in relation with a decreased number of Purkinje cells (Sajdel-Sulkowska et al., 2005) that could be related to a transient hypothyroidism induced by hypergravity (Sajdel-Sulkowska et al., 2001, 2005). Other analyses carried out during limited parts of the development showed two periods more sensitive to hypergravity: the second gestational week that coincides with the period of Purkinje cells birth, and the 2nd and 3rd postnatal weeks that coincide with the peak in granule cell neurogenesis (Nguon et al., 2006b). The alteration of cerebellar development in centrifuged rats could be related to a change in the quantitative or temporal expression of proteins involved in cell-cell interaction (Sajdel-Sulkowska, 2008).

### Descending pathways

At the level of the spinal cord, hypergravity induced a delay in the development of descending pathways, including reticulo- and vestibulo-spinal tracts, (Brocard et al., 2003) and a hyperactivity of lumbar motoneurons (Krasnov et al., 1992). The development in hypergravity also provoked a substantial delay of the development and strong perturbations of monoaminergic projections to the spinal cord in newborn rats centrifuged (1.8G) from E10 to PND15. An anarchic pattern of innervation, with numerous dystrophic profiles mainly of serotonergic system, was present at PND 15 and persisted in 8 months old animals, suggesting that rats submitted to hypergravity during the critical period of onset of monoaminergic projections to the spinal cord are affected durably in the organization and the ultrastructure of these projections (Gimenez y Ribotta et al., 1998). Neonate rats flown postnatally in a spaceflight from PND8 to PND24 showed a reduced development of dendritic trees in a population of motor neurons innervating axial and proximal muscles (Inglis et al., 2000). This observation suggested a possible reduction of the synaptic activation of these motor neurons due to the hypo activation of the otolithic system in microgravity.

These studies suggested that altering gravity perturb the connectivity of vestibulo-spinal pathways. Together these results suggest that the motor development was delayed when the gravity sensing system was either overstimulated or deprived. They demonstrate that the environment plays a critical role in fine-tuning of axons, and for appropriate development of the projections from gravity receptors to the brain and spinal cord.

### Cognition

The possible consequences of the early exposure to altered gravity on the cognitive development is a pending question because cognitive alterations have been reported in adults exposed to altered gravity. There are two main ways of action of gravity on the cognitive functions. On the one hand, the organization of somatotopic maps is strongly influenced by sensory experience early in life and to a lesser extent in adulthood. The somato-topic maps in primary somatosensory cortex constitute neural networks which play a pivotal role in sensory integration and perceptual learning. Long-lasting changes in the properties of somatosensory cortical neurons and discrimination abilities have been reported after early alteration in tactile experience (Coq and Xerri, 2001). Hypergravity or microgravity change the representations of muscles in the somatosensory cortex of adult rats (D'amelio et al., 1998a,b; Trinel et al., 2013). The durable consequences of early motor experience in altered gravity on the somatosensory maps are unknown, but a study on rats flown in the space station for 16 days from PND14 to PND30 showed durable changes even after 4 months (Defelipe et al., 2002). On the other hand, there is a convergent opinion that changes in peripheral and central vestibular neurotransmission contribute to the impaired spatial learning through decreased vestibular inputs to areas important for spatial cognition such as the hippocampus. The hypothesis of a role of vestibular inputs on spatial cognition is supported by the demonstrations that loss of vestibular function alters spatial cognition (Brandt et al., 2005; Ventre-Dominey et al., 2005; Smith et al., 2010). A possible explanation for the role of vestibular input on the impairment of spatial learning could be related to a mismatch of the otolithic contribution to the head direction cells (Stackman and Taube, 1997; Taube, 2007; Yoder and Taube, 2009). The influence of gravity on the spatial impairment is supported by changes in the expression of hippocampal genes specifically modulated by hypergravity (Del Signore et al., 2004) or microgravity (Santucci et al., 2012), but changes in the gene expression in the hippocampus was observed also in tail suspended mice, a situation that do not alter gravity sensing (Sarkar et al., 2006). The specific contribution of the altered gravity in the gene expression of the hippocampus is therefore always questionable. Alternately altered cognition and the change of genes expression in the hippocampus could reflect changes in the brain-hypothalamic-pituitary-adrenal axis due to the chronic stressful experience, involving a stressor specifically associated to hypergravity (Del Signore et al., 2004). This hypothesis is supported by the existence of a hypothalamic-pituitary-adrenal axis activation induced by vestibular lesions (Gliddon et al., 2003a,b). Gravity changes could also alter cognitive function via modulation of brain vascular reactivity (Porte and Morel, 2011). In addition, high levels of hypergravity induce severe impairments in cognitive abilities which may be associated with brain ischemia (Sun et al., 2009). An influence of hypergravity on the performance of adult rats and mice in spatial memory tests was observed after chronic centrifugation (2G) for 2 weeks (Mitani et al., 2004) or after repeated episodes of 1 h of centrifugation (1.85G) during 5 days (Mandillo et al., 2003), or after short exposure (3 min) to high level of hypergravity (6G) (Cao et al., 2007; Sun et al., 2009). The information about the effect of altered gravity during the development of the cognitive functions was rarely studied. Except for the exposure of peri-adolescent mice to short episodes of acute centrifugation (Francia et al., 2004; Santucci et al., 2009), the unique analysis of cognitive function in rats flown postnatally between PND 14 and 31 did not show any difference in spatial orientation or brain structure (Temple et al., 2002).

## Vestibular reactions

The evaluation of vestibular efficiency in neonate rodents is mainly based on the observation of righting reflex. The righting from a supine to a prone position is a basic motor pattern that rat and mice pups are able to perform on the first postnatal day (Roubertoux et al., 1985; Pellis et al., 1991), because it is necessary to reach the nipple (Eilam and Smotherman, 1998). Testing the righting reflex is done by means of contact righting, or water immersion, or air righting, to avoid proprioceptive information to aid the response. The righting response involves the proper dynamic interaction of otolith dependent vestibulo-colic and vestibulo-spinal reflexes, and otoconia deficient mice are unable to perform righting response.

### Microgravity

A few space missions allowed either a prenatal or a postnatal exposure to microgravity. Rats flown in a space flight prenatally (E8–E19) showed an early righting impairment that was recovered from PND5 (Ronca and Alberts, 1997, 2000; Ronca et al., 2000, 2008). Eventually, they did not show any change on successive timing of hind limb motor development over a period of 81 days after landing (Wong and Desantis, 1997). Rats exposed postnatally to the space environment (from PND15 to PND24 or from PND14 to PND 30) were able to swim (Temple et al., 2002; Walton et al., 2005a), and to perform surface righting at landing time. This result is contradictory with the inability to float of otoconia-deficient mice and suggest that the vestibular apparatus and vestibulo-colic reflexes were functional within hours of landing. The vestibular information required for this reflex was therefore not altered by microgravity. Nevertheless postnatally flown rats showed impaired maturation in the acquisition of adult tactics of surface righting, and the pups flown until PND30 were definitely unable to perform adult tactics for surface righting (Walton et al., 2005b). For the authors the impairment was probably not due to a sensory deficit, but rather to the missing acquisition of the correct motor pattern during a critical period of motor development. Other long term modifications of motor parameters were observed after the postnatal exposure to microgravity (Walton et al., 1992, 2003, 2005a; Walton, 1998), but they are supposed to be related to strong consequence of gravity on the muscles properties and muscle representation in the brain (Defelipe et al., 2002). They are not considered here as direct consequences of an alteration of gravity sensing. The exposure to microgravity seemed to cause a delay in the acquisition of vestibular reactions during prenatal development, whereas the consequences on postnatal development concerned the acquisition of motor skills. Even though they provided relevant results, the conclusions of these experiments are minored by interrogations concerning the good fit of the flight opportunity with potential critical periods. Ronca et al. (2008) suggested that exposure to microgravity throughout the entirety of neurovestibular development likely produce irreversible or at least enduring deficit in vestibular response. Unfortunately the technical limitations of spaceflight missions do not allow to expose rodents for large part of their development.

### Hypergravity

Several experiments exposed mammals to hypergravity during the complete maturation of the vestibular system. A first experiment centrifuged hamsters from conception to at least 4 weeks and showed reduced performance in swimming ability and air righting that prolonged after months, particularly in hamsters centrifuged until 20th postnatal week. These results were interpreted to reflect a dysfunction in the otolithic system (Sondag et al., 1997). Later studies on rats did not confirm this result. A similar experiment performed on rats did not show differences between hypergravity and control rats (Wubbels and De Jong, 2000), but experimental artifacts possibly interfered. In another study, rats centrifuged (1.75G) from E8 to PND21 showed a poor score on the rotarod at PND21 (Nguon et al., 2006a). The authors correlated this bad performance with a decreased cerebellar mass. Other experiments on rats centrifuged (1.8G) from conception to PND21 or PND27 showed a delay in the vestibular reflexes that persisted at PND40 (Bouet et al., 2004b). Another study on rats centrifuged until the age of 3 months showed that a complete behavioral recovery occurred in a delay of 3 weeks (Bouet et al., 2003, 2004a). Mice centrifuged (2G) from conception to PND30 showed a delay in the acquisition of maculo-ocular reflex (Beraneck et al., 2012). At the age of 2 months they were not impaired in vestibular response, but showed a tendency to react slower during free fall that was supposed to result from a perturbation of the connections between vestibular, cerebellar and motor structures (Bojados and Jamon, 2012). Together the hypergravity studies suggested that centrifuging during the full development induced transitory vestibular impairment and a possible alteration of vestibulo-cerebellar connections, but the effects were not as drastic in rat and mice than in hamsters. That discrepancy lets open two possibilities: (1) the persistent impairment in hamsters was related to a different development in cricetidae, although the time schedule of development is rather similar to the rats. (2) detrimental effects of the exposure to hypergravity on the vestibular system could be caused by excitoxicity in relation with the duration of exposure (Beraneck et al., 2012).

### Prenatal centrifugation

Rats centrifuged (1.8G) from conception to PND10 showed a slight delay in the acquisition of vestibular reflexes that was restored within 21 days (Bouet et al., 2004b). Mice centrifuged during the same period and tested at the age of 2 months were not perturbed in the vestibular tests, and even tended to improve their performance. In addition they showed specific improvement of their aerobic capacities, and a change in postural parameters (Bojados and Jamon, 2012; Bojados et al., 2013). These results showed that vestibular performance was not definitely affected by a prenatal exposure to hypergravity, whereas other changes were definitive. When centrifugation of rats was limited to the second or third gestational week, only the former period induced an impaired equilibrium performance on the rotarod at PND21. This result suggested a possible critical period during the second gestational week in relation with the birth of Purkinje cells at E13–E14 (Nguon et al., 2006b).

### Postnatal centrifugation

Postnatal exposure to hypergravity was rarely studied, but rats centrifuged during the second and third weeks of postnatal development showed the more critical sensitivity to hypergravity exposure (Nguon et al., 2006b), as showed the worst performance on the rotarod at PND21. This postnatal period corresponds to a peak in granule neurogenesis, a period that appears to be critical with respect to the maturation of cerebellar structure and function. Mice centrifuged (2G) from PND10 to PND30 were not significantly impaired in the maculo-ocular reflex (Beraneck et al., 2012), but they showed slower vestibular reactions during the drop tests, in addition to changes in the motor pattern (Bojados et al., 2013). The experiments on postnatal exposure to hypergravity suggest that long term impairment was probably related to the connection between vestibular and cerebellar and motor structure.

## Studies on vestibular deficient mutant mice

Alternative strategies using ground-based experiments take advantage of the development of targeted mutations in mice. More than 25 lines of mice with congenital vestibular mutations were available in 2002 (Anagnostopoulos, 2002), and the number is increasing. Vestibular deficient mice with a null mutation of the KCNE1 potassium-channel gene that leads to the degeneration of hair cells (Vetter et al., 1996) show a permanent shaker/waltzer phenotype (Vidal et al., 2004) that is caused by dopamine asymmetry due to the absence of vestibular input in the striatum during critical period of the development. The complete removal of vestibular organs before PND5 leads to permanent head bobbing in adults (Geisler et al., 1996; Geisler and Gramsbergen, 1998). This phenomenon was related to the lack of semi-circular canals input rather than the lack otolithic afference (Eugene et al., 2009). Other mutations affect more specifically the graviceptors. Several lines of mice have specific alteration of genes involved in the formation of the otoconia while other components of the vestibular system are intact (Ornitz et al., 1998). The shaker/waltzer phenotype is not observed in these otoconia-deficient mice. They have a typically permanent head tilt phenotype that could be the signature of an otoconial critical period. Studies of mouse strains with graded otoconial deficiencies showed graded loss of function, and confirmed the absence of linear acceleration vestibular evoked potentials in mice lacking completely otoconia (Jones et al., 2004). Morphometric analyses of the vestibular ganglia performed at various stages of the postnatal development of *tilted* mice showed a slower development during the first postnatal week (either rate of development or cell number), then they reached values similar to control, and eventually possess normal appearing sensory epithelia. The absence of gross abnormality in the vestibular ganglia may be due to the spontaneous activity of receptor cells which maintain tonic stimulation of the ganglionic cells (Smith et al., 2003). It was demonstrated that the presence of otoconia is not required for the general formation and maintenance of synapses (Hoffman et al., 2006) or normal development of vestibular ganglia (Smith et al., 2003). Mutant mice, with the tilted mutation (*tlt*) that eliminates an essential component necessary for the formation of otoconia, can learn gravity dependent motor task by using semi-circular canals and limb proprioception to compensate otoconia deficiency (Crapon De Caprona et al., 2004). However head tilt (*het*) mice with a recessive mutation causing a complete lack of otoconia are unable to perform task requiring equilibrium or postnatal reflexes and do not perform righting response. They show also an alteration of working spatial memory and place recognition (Machado et al., 2012), that is supposed to be originating from an abnormal modulation of head direction cells.

There are major limitations with the use of mutant lines to evaluate the consequences of specific alterations of the vestibular apparatus, because most mutants may have potentially compromised hair cells, stereocilia or vestibular ganglia, due to the expression of mutated genes in these structures. In addition the definitive removal of graviception is susceptible to produce compensation mechanisms (Crapon De Caprona et al., 2004). For instance, in the *IED* (Inner Ear Defect) mice, in the absence of otolothic information, visual inputs become instrumental for gaze stabilization (Beraneck et al., 2012). Nevertheless the generation of inducible conditional knockout mice, which allows selected inactivation of genes in tissues at a given time point, is of prime importance to complement micro- and hypergravity studies.

## The hypothesis of critical periods in the adaptation to gravity

A survey of the literature covering the last decade shows that many of the consequences of the exposure to altered gravity during the development of vestibular sensitivity were supposed to involve a critical period. The existence of critical period was invoked about the development of gravity sensing concerning the peripheral organ (otoconia, sensory epithelium), the vestibular nuclei, the cortical projections, the cerebellar connections, and the motor output. In most cases the critical periods were hypothesized when a definitive or at least a long lasting change was observed after the exposure to altered gravity during some developmental stages. The long term duration of the change is not mandatory and some authors prefer to use the term of sensitive period. In addition, further events occurring during the life history are susceptible to reduce or to mask the changes induced during a critical period (Bojados and Jamon, 2012). A more precise assessment of the meaning of critical period have an heuristic value for understanding the consequences of the exposure to altered gravity on developing structures.

A critical period is a time window of the early life when the experience of external information is needed for the normal development of a structure or a function. The brain needs this external sensitivity period to tune the receptor with the source when precise information about the individual or the environment cannot be predicted and therefore cannot be genetically encoded. The critical period corresponds therefore to an interactive specialization in the functional organization of brain regions or cortical areas. A critical period can only open when the structure concerned has achieved its embryological development. GABAergic neurons have a main role in the internal control of critical period timing. For instance the onset and duration of visual critical period is advanced in mice over expressing BDNF, that accelerate maturation of GABAergic neurons (Huang et al., 1999). At variance the critical period for ocular dominance is inhibited in mice lacking Gad65 gene, that show poor GABA release, and is restored with diazepam which acts as a GABA agonist (Hensch et al., 1998).

In addition to the internal process, relevant sensory information is required, and the critical period can be delayed and prolonged to some extent when the information is not available. Dark rearing, for instance, delays the maturation of GABAergic transmission and the onset of visual critical period, but BDNF supplementation abolishes this delay (Hensch and Fagiolini, 2005). The termination of the critical period is a consequence of the mechanisms by which the cortical regions become increasingly specialized and fine-tuned. Changes in the brain neurochemistry as for instance the composition of N-Methyl-D-Aspartate (NMDA) receptor increase the rate of pruning of synapses and results in a freezing of the pattern of functional sensitivity.

The achievement of the critical periods involves three main stages (Hensch, 2004): (1) expansion of axonal branching and synapses formation in association with a high level of grow associated proteins and neurotrophic factors, particularly BDNF. With this process neurones invade narrow brain regions and elaborate new projection fields, the response properties of cortical regions interact and compete to acquire their role in new abilities; (2) a further shaping of the circuit architectures is realized by pruning less solicited axons and synapses, on the basis of the competition between neural inputs on common targets. The structural consequences of the functional competition for brain regions was illustrated by the lateral dominance occurring in the cortical mapping of sensory entries after hemi deprivation of vision, audition or somatosensory system; (3) structural stabilization of potentiated synapses by the insertion of cell adhesion molecules, change in the composition of NMDA receptor and limitation of GABAergic large basket cells in an extracellular matrix. The consolidated synapses become invulnerable to further elimination and make further plasticity harder to occur.

On the basis of the theoretical process of critical period, two main consequences are expected from the lack of gravity sensing during the development in microgravity: (1) a delayed maturation is expected because the onset of critical period is prevented by the unavailability of the sensory information. This should leave immature characteristics in the system; (2) A colonisation of gravity related vestibular projections by competing afferences, particularly the expansion of inputs from the angular acceleration detectors which continue to be stimulated in microgravity. These expected consequences are consistent with the delayed maturation reported in rats flown prenatally. For instance, rats exposed to microgravity during the second half of the gestational development showed a delay in the synaptogenesis of saccular neurones and an increased proportion of angular acceleration synapses (Ronca et al., 2008). These observations were supposed to involve an overstimulation of canalar inputs *in utero* due to the 3D movements of the floating dam (Ronca et al., 2008), but they are also consistent with delayed development and competition for neural branching, and therefore support the existence of a critical period. In the same way the possibility was evoked for otolithic deficient *IED* mice that in the absence of gravity related signals central vestibular neurons would substitute otolith inputs with spatially non matching canal inputs (Beraneck et al., 2012).

Hypergravity can be considered as a mirror situation to microgravity (Serova et al., 1985; Serova, 1991; Phillips, 2002; Wade, 2005). From the point of view of critical period, the situation is not symmetrical however. Obviously sensory deprivation does not apply to this situation. In the hypergravity environment, the gravitational stimulus is not removed, but increased instead, producing therefore an overstimulation with reference to the other stimuli. It is important to distinguish between overstimulation, under stimulation and deprivation. Nevertheless two logical consequences of the concept of critical period apply to hypergravity: (1) A possible faster maturation instead of a delay in maturation due to the hyper stimulation; (2) The expansion of gravity related pathways at the detriment of competing afferences. From this point of view, it is noteworthy that various effects observed in rats developed in hypergravity were associated with faster rates of embryo maturation (Bruce et al., 2006). Indeed several pieces of information seem to comfort the advanced maturation of the peripheral organ when exposed to hypergravity during the morphological development of structures. On the other hand other studies concluded to a retarded development and the persistence of impairments. The interaction of multiple structures, each with different time schedule, produces a complex situation when the developing organisms are exposed to hypergravity, and induces potential factors of trouble at the origin of this dichotomy. Four of them are listed below:

1. The advanced maturation of the vestibular apparatus, that can be theoretically expected in case of overstimulation during a critical period, is susceptible to perturb the synchrony with later developing structures, as for instance the cell-cell interaction between vestibular and cerebellar structures. Note that reciprocal consequence apply to the delayed development of vestibular structures in microgravity. This could explain why both micro- and hypergravity induce a perturbation in vestibular-related structures.
2. The sensory system adapts to the over intensity of the gravity signal by reducing the otoconia mass, the connections in the maculae, and the hair cells responses. Consequently, the vestibular apparatus is under tuned for Earth gravity. This could be another source of dysfunction during the establishment of connections with other structures, and then could participate to the impairment of the vestibulo-cerebellar or vestibulo-motor functions.
3. Due to the competition of sensory entries for neural targets during the critical periods, the overstimulated otolith structures should be overrepresented in vestibular nuclei and brain targets, at the detriment of the canalar afferences. This disequilibrium is a potential source of trouble because the otolithic and canalar afferences are heavily mixed to contribute to the vestibular signal.
4. The prolonged exposure to hypergravity could have detrimental effects on the vestibular system by excitotoxicity due to the overstimulation, as occurs for other sensory entries (Peusner, 2001). This effect should be dependent on the duration and probably of the intensity of the overstimulation, and could explain some long term deficiency observed after a long period of centrifugation.

## The vestibular development in precocial or altricial species

The analysis of vestibular development refers either to prenatal or postnatal exposure to altered gravity. The consecutive differences are basically related to the development of the vestibular apparatus, that is mainly prenatal, and vestibular-related functions that develop heavily after birth. The vestibular and motor immaturity contribute to the unachieved postural and motor control observed in rats and mice (Clarac et al., 1998; Muir, 2000), even though behavioral adaptations contribute to the silencing of motor activities in nesting mammals (Jamon, 2006). The immaturity of postural control at birth opens speculations about a possible role of the experience *ex utero* for the maturation of postural control. At variance, precocious species are relatively mature and mobile at birth and acquire rapidly the adult-like motor control. Typically altricial species have poorly developed offspring, with eyes and ears closed at birth, virtually no hairs on the body, and are typically born in multiple litters, whereas precocial species have well developed offspring with eyes and ears open at (or soon after) birth, hair coat well developed, and are typically born as singletons. Altricial and precocial mammals exhibit no difference in the rate of growth (Case, 1978), and show similar trend course of neural development (Clancy et al., 2001), with the only difference being the arbitrary point of birth (Brunjes, 1988). The longer gestation period in precocial species results in increased development of the central nervous system at birth (Sacher and Staffeldt, 1974). The duration of gestation is therefore the main factor of maturity at birth. The comparative development of guinea pig and rat shows a typical example of the difference in the level of maturity at birth. In guinea pig the gestation lasts 66 days on the average. General movements emerge between E24 and E34 in guinea pig fetuses. The period of E35–E40 is characterized by an established link between the vestibular apparatus and vestibular ganglia (Heywood et al., 1976; Sobin and Anniko, 1983), and myelination of vestibular nerve begins about E40. Cortical differentiation occurs between E41 and E45 (Van Kan et al., 2009). The righting reflex develop *in utero* between E50 and E66 (Sekulic et al., 2009). Standing and walking at present from E63 (Avery, 1928). The motor abilities of precocial species suggest that vestibular functions have matured *in utero*. Even though intrauterine cavity is similar to a microgravity environment (Wood, 1970; Sekulic et al., 2005; Meigal, 2013) because of neutral buoyancy in the amniotic fluid, the otoliths in the intrauterine environment are constantly exposed to the effects of gravity, and are stimulated by the linear accelerations induced by the movements of the mother (Ronca et al., 1993). In addition hair cells do not need the gravitational stimulus to develop. Therefore postnatal experience is no necessary for the system to mature. The process of postnatal maturation in altricial species is therefore independent of vestibular-driven afference. Nevertheless the system continues to develop in precocial species, as well as in altricial species in relation with increasing hair cells number (Jones and Jones, 2000) and the vestibulo-ocular reflex in relation with the increasing size of canals (Straka, 2010). This supports the possibility that the vestibular system is finely tuned with the development of posturo-motor functions. Developing young mice are subjected to direct and indirect effects when exposed to altered gravitational field (Alberts and Ronca, 2005). Among them, the high level of interaction between the mother and pups, particularly in altricial species, is susceptible to be perturbed. The mother and pups constitute a “maternal-offspring system” of paramount importance for the proper development of the young (Ronca, 2003; Alberts and Ronca, 2005) by directing and regulating postnatal development. Licking and grooming are important sources of stimulation that contribute to the brain development. These maternal cares stimulate the expression of BDNF and other neural systems (Curley et al., 2011) in brain parts, resulting in improved emotionality, sociality and learning abilities of pups (Caldji et al., 1998; Liu et al., 2000; Branchi et al., 2013), and their perturbation is detrimental for cognitive development. Given the importance of BDNF, GABA and glutamate in the regulation of critical periods, the consequences of altered gravity on the maternal care deserve to be investigated further. This aspect could be investigated by means of comparative developmental studies involving altricial (rat and mice) and precocial (Acomys, mesocricetus) species subjected to altered gravity.

## Conclusions

The present review showed accumulating evidence on the sensitivity of the organisms to the alteration of gravity during their development. The peripheral sensory organ adapts to the level of gravity by adjusting the mass of otoconia and the innervation of sensory epithelium. The over or under stimulation advances or delays the maturation of neural connections during the formation of the vestibular apparatus, resulting in inadequate temporal synchrony or sensibility tuning during the connections with vestibular-related structures, and with potential long-term change in the resulting functions. These results provide further evidence that the gravistatic sensory system has a genetically controlled phase of development for target finding and a stimulus-controlled phase for fine-tuning synaptic terminals. Therefore the level of gravity plays a critical role in fine-tuning of axons and is required for appropriate development of the projections from graviceptors to the brain and spinal cord. Several critical periods for the adaptation to gravity are probably spread along the developmental process, in relation with the timing of the various structures involved, and with variable incidence depending on the plasticity of the structures. The time windows of possible critical periods in the development of the various structures can be hardly answered due to the difficulty to remove gravity vector from Earth environment and the limited access to space missions for rodent studies. This question could be answered with long duration space flight as promised the ISS, but does not seem realistic at present. Facing these difficulties the development of ground based techniques becomes necessary. The use of centrifugation to produce hypergravity is potentially a useful tool to detect the critical periods, provided that a careful attention is given to the expected criteria to detect a critical period when the stimulus if amplified instead of removed. In addition, a standardization of the centrifugation techniques should be desirable. On the other hand the use vestibular deficient mutated mice proved to be useful, and the availability of conditioned KO is a promising tool for the future.

### Conflict of interest statement

The author declares that the research was conducted in the absence of any commercial or financial relationships that could be construed as a potential conflict of interest.

## References

[B1] Alberts J. R. Ronca A. E. (2005). Development as adaptation: a paradigm for gravitational and space biology, in Experimentation with Animal Models in Space. Advances in space biology and Medecine, ed Sonnenfeld G. (Elsevier), 175–207 10.1016/S1569-2574(05)10007-0 16101108

[B2] Alberts J. R. Serova L. V. Keefe J. R. Apanasenko Z. (1985). Early postnatal development of rats gestated during flight of Cosmos 1514. Physiologist 28, S81–S82 3834496

[B3] Anagnostopoulos A. V. (2002). A compendium of mouse knockouts with inner ear defects. Trends Genet. 18, 499 10.1016/S0168-9525(02)02753-1 12350347

[B4] Anken R. H. Beier M. Edelmann E. Rahmann H. (2002a). Neuronal regulation of otolith growth and kinetotic behaviour. J. Gravit. Physiol. 9, P37–P38 14703676

[B5] Anken R. H. Beier M. Rahmann H. (2002b). Influence of hypergravity on fish inner ear otoliths: I. Developmental growth profile. Adv. Space Res. 30, 721–725 10.1016/S0273-1177(02)00389-7 12528670

[B6] Anken R. H. Ibsch M. Rahmann H. (2002c). Microgravity (STS-90 Neurolab-Mission) influences synapse formation in a vestibular nucleus of fish brain. Adv. Space Res. 30, 843–847 10.1016/S0273-1177(01)00643-3 12530421

[B7] Anken R. H. Ibsch M. Breuer J. Rahmann H. (2001). Effect of hypergravity on the Ca/Sr composition of developing otoliths of larval cichlid fish (*Oreochromis mossambicus*). Comp. Biochem. Physiol. A Mol. Integr. Physiol. 128, 369–377 10.1016/S1095-6433(00)00316-0 11223398

[B8] Anniko M. (1980). Development of otoconia. Am. J. Otolaryngol. 1, 400–410 10.1016/S0196-0709(80)80021-4 7457761

[B8a] Avery G. T. (1928). Responses of foetal guinea pigs prematurely delivered. Genet. Psychol. Monogr. 3, 245–331

[B9] Beisel K. W. Wang-Lundberg Y. Maklad A. Fritzsch B. (2005). Development and evolution of the vestibular sensory apparatus of the mammalian ear. J. Vestib. Res. 15, 225–241 16614470 PMC3901525

[B10] Beraneck M. Bojados M. Le Seac'h A. Jamon M. Vidal P. P. (2012). Ontogeny of mouse vestibulo-ocular reflex following genetic or environmental alteration of gravity sensing. PLoS ONE 7:e40414 10.1371/journal.pone.0040414 22808156 PMC3393735

[B11] Bojados M. Herbin M. Jamon M. (2013). Kinematics of treadmill locomotion in mice raised in hypergravity. Behav. Brain Res. 244, 48–57 10.1016/j.bbr.2013.01.017 23352767

[B12] Bojados M. Jamon M. (2012). Exposure to hypergravity during specific developmental periods differentially affects metabolism and vestibular reactions in adult C57BL /6j mice. Eur. J. Neurosci. 34, 2024–2034 10.1111/j.1460-9568.2011.07919.x 22122506

[B13] Bouet V. Borel L. Harlay F. Gahery Y. Lacour M. (2004a). Kinematics of treadmill locomotion in rats conceived, born, and reared in a hypergravity field (2 g). Adaptation to 1 g. Behav. Brain Res. 150, 207–216 10.1016/S0166-4328(03)00258-4 15033294

[B14] Bouet V. Wubbels R. J. De Jong H. A. Gramsbergen A. (2004b). Behavioural consequences of hypergravity in developing rats. Brain Res. Dev. Brain Res. 153, 69–78 10.1016/j.devbrainres.2004.03.022 15464219

[B15] Bouet V. Gahery Y. Lacour M. (2003). Behavioural changes induced by early and long-term gravito-inertial force modification in the rat. Behav. Brain Res. 139, 97–104 10.1016/S0166-4328(02)00085-2 12642180

[B16] Branchi I. Curley J. P. D'Andrea I. Cirulli F. Champagne F. A. Alleva E. (2013). Early interactions with mother and peers independently build adult social skills and shape BDNF and oxytocin receptor brain levels. Psychoneuroendocrinology 38, 522–532 10.1016/j.psyneuen.2012.07.010 22910688 PMC3522751

[B17] Brandt T. Schautzer F. Hamilton D. A. Bruning R. Markowitsch H. J. Kalla R. (2005). Vestibular loss causes hippocampal atrophy and impaired spatial memory in humans. Brain 128, 2732–2741 10.1093/brain/awh617 16141283

[B18] Brocard F. Clarac F. Vinay L. (2003). Gravity influences the development of inputs from the brain to lumbar motoneurons in the rat. Neuroreport 14, 1697–1700 10.1097/00001756-200309150-00008 14512840

[B19] Brocard F. Vinay L. Clarac F. (1999). Development of hindlimb postural control during the first postnatal week in the rat. Dev. Brain Res. 117, 81–89 10.1016/S0165-3806(99)00101-7 10536235

[B20] Bruce L. L. (2003). Adaptations of the vestibular system to short and long-term exposures to altered gravity. Adv. Space Res. 32, 1533–1539 10.1016/S0273-1177(03)90392-9 15000124

[B21] Bruce L. L. Burke J. M. Dobrowolska J. A. (2006). Effects of hypergravity on the prenatal development of peripheral vestibulocerebellar afferent fibers. Adv. Space Res. 38, 1041–1051 10.1016/j.asr.2006.03.002

[B22] Bruce L. L. Fritzsch B. (1997). The development of vestibular connections in rat embryos in microgravity. J. Gravit. Physiol. 4, P59–P62 11540700

[B23] Brugeaud A. Gaboyard-Niay S. Puel J. L. Chabbert C. (2006). Hypergravity affects the developmental expression of voltage-gated sodium current in utricular hair cells. Neuroreport 17, 1697–1701 10.1097/01.wnr.0000239961.98813.19 17047456

[B24] Brunjes P. C. (1988). Precocity and plasticity: odor deprivation and brain development in the precocial mouse *Acomys cahirinus*. Neuroscience 24, 579–582 10.1016/0306-4522(88)90351-X 3362353

[B25] Caldji C. Tannenbaum B. Sharma S. Francis D. Plotsky P. M. Meaney M. J. (1998). Maternal care during infancy regulates the development of neural systems mediating the expression of fearfulness in the rat. Proc. Natl. Acad. Sci. U.S.A. 95, 5335–5340 10.1073/pnas.95.9.5335 9560276 PMC20261

[B26] Cao X.-S. Sun X.-Q. Zhang S. Wang B. Wu Y.-H. Liu T.-S. (2007). Acceleration after-effects on learning and memory in rats: +10 Gz or +6 Gz for 3 min. Neurosci. Lett. 413, 245–248 10.1016/j.neulet.2006.11.055 17175101

[B27] Case T. J. (1978). On the evolution and adaptive significance of postnatal growth rates in the terrestrial vertebrates. Q. Rev. Biol. 53, 243–282 10.1086/410622 362471

[B28] Chizhikov V. Millen K. J. (2003). Development and malformations of the cerebellum in mice. Mol. Gen. Metabol. 80, 54–65 10.1016/j.ymgme.2003.08.019 14567957

[B29] Clancy B. Darlington R. B. Finlay B. L. (2001). Translating developmental time across mammalian species. Neuroscience 105, 7–17 10.1016/S0306-4522(01)00171-3 11483296

[B30] Clarac F. Vinay L. Cazalets J. R. Fady J. C. Jamon M. (1998). Role of gravity in the development of posture and locomotion in the neonatal rat. Brain Res. Brain Res. Rev. 28, 35–43 10.1016/S0165-0173(98)00024-1 9795120

[B31] Coq J. O. Xerri C. (2001). Sensorimotor experience modulates age-dependent alterations of the forepaw representation in the rat primary somatosensory cortex. Neuroscience 104, 705–715 10.1016/S0306-4522(01)00123-3 11440803

[B32] Crapon De Caprona M. D. Beisel K. W. Nichols D. H. Fritzsch B. (2004). Partial behavioral compensation is revealed in balance tasked mutant mice lacking otoconia. Brain Res. Bull. 64, 289–301 10.1016/j.brainresbull.2004.08.004 15561463

[B33] Curley J. P. Jensen C. L. Mashoodh R. Champagne F. A. (2011). Social influences on neurobiology and behavior: epigenetic effects during development. Psychoneuroendocrinology 36, 352–371 10.1016/j.psyneuen.2010.06.005 20650569 PMC2980807

[B34] Curthoys I. S. (1979a). The development of function of horizontal semicircular canal primary neurons in the rat. Brain Res. 167, 41–52 10.1016/0006-8993(79)90261-0 455071

[B35] Curthoys I. S. (1979b). The vestibulo-ocular reflex in newborn rats. Acta Otolaryngol. 87, 484–489 10.3109/00016487909126456 313655

[B36] D'amelio F. Fox R. A. Wu L. C. Daunton N. G. Corcoran M. L. (1998a). Effects of microgravity on muscle and cerebral cortex: a suggested interaction. Adv. Space Res. 22, 235–244 11541401 10.1016/s0273-1177(98)80015-x

[B37] D'amelio F. Wu L.-C. Fox R. A. Daunton N. G. Corcoran M. L. Polyakov I. (1998b). Hypergravity exposure decreases γ-aminobutyric acid immunoreactivity in axon terminals contacting pyramidal cells in the rat somatosensory cortex: a quantitative immunocytochemical image analysis. J. Neurosci. Res. 53, 135–142 9671970 10.1002/(SICI)1097-4547(19980715)53:2<135::AID-JNR2>3.0.CO;2-8

[B38] Dechesne C. Mbiene J. P. Sans A. (1986). Postnatal development of vestibular receptor surfaces in the rat. Acta Otolaryngol. 101, 11–18 10.3109/00016488609108602 3485880

[B39] Defelipe J. Arellano J. I. Merchan-Perez A. Gonzalez-Albo M. C. Walton K. Llinas R. (2002). Spaceflight induces changes in the synaptic circuitry of the postnatal developing neocortex. Cereb. Cortex 12, 883–891 10.1093/cercor/12.8.883 12122037

[B40] Del Signore A. Mandillo S. Rizzo A. Di Mauro E. Mele A. Negri R. (2004). Hippocampal gene expression is modulated by hypergravity. Eur. J. Neurosci. 19, 667–677 10.1111/j.0953-816X.2004.03171.x 14984417

[B41] Dememes D. Dechesne C. J. Venteo S. Gaven F. Raymond J. (2001). Development of the rat efferent vestibular system on the ground and in microgravity. Brain Res. Dev. Brain Res. 128, 35–44 10.1016/S0165-3806(01)00146-8 11356260

[B42] Denise P. Besnard S. Vignaux G. Sabatier J. P. Edy E. Hitier M. (2009). Sympathetic B antagonist prevents bone mineral density decrease induced by labyrinthectomy. Aviakosm. Ekolog. Med. 43, 36–38 20169738

[B43] Desmadryl G. (1991). Postnatal developmental changes in the responses of mouse primary vestibular neurons to externally applied galvanic currents. Brain Res. Dev. Brain Res. 64, 137–143 10.1016/0165-3806(91)90217-7 1786638

[B44] Desmadryl G. Raymond J. Sans A. (1986). *In vitro* electrophysiological study of spontaneous activity in neonatal mouse vestibular ganglion neurons during development. Brain Res. 390, 133–136 10.1016/0165-3806(86)90160-4 3948026

[B45] Desmadryl G. Sans A. (1990). Afferent innervation patterns in crista ampullaris of the mouse during ontogenesis. Brain Res. Dev. Brain Res. 52, 183–189 10.1016/0165-3806(90)90234-P 2331787

[B46] Eilam D. Smotherman W. P. (1998). How the neonatal rat gets to the nipple: common motor modules and their involvement in the expression of early motor behavior. Dev. Psychobiol. 32, 57–66 10.1002/(SICI)1098-2302(199801)32:1<57::AID-DEV7>3.0.CO;2-S 9452909

[B47] Eugene D. Deforges S. Vibert N. Vidal P. P. (2009). Vestibular critical period, maturation of central vestibular neurons, and locomotor control. Ann. N.Y. Acad. Sci. 1164, 180–187 10.1111/j.1749-6632.2008.03727.x 19645897

[B48] Fiez J. A. (1996). Cerebellar contributions to cognition. Neuron 16, 13–15 10.1016/S0896-6273(00)80018-5 8562076

[B49] Francia N. Santucci D. Chiarotti F. Alleva E. (2004). Cognitive and emotional alterations in periadolescent mice exposed to 2 g hypergravity field. Physiol. Behav. 83, 383–394 10.1016/j.physbeh.2004.08.011 15581660

[B50] Freeman S. Plotnik M. Elidan J. Sohmer H. (1999). Development of short latency vestibular evoked potentials in the neonatal rat. Hear. Res. 137, 51–58 10.1016/S0378-5955(99)00137-9 10545633

[B51] Fritzsch B. (2003). Molecular developmental neurobiology of formation, guidance and survival of primary vestibular neurons. Adv. Space. Res. 32, 1495–1500 10.1016/S0273-1177(03)90387-5 15000110

[B52] Fritzsch B. Beisel K. W. Jones K. Farinas I. Maklad A. Lee J. (2002). Development and evolution of inner ear sensory epithelia and their innervation. J. Neurobiol. 53, 143–156 10.1002/neu.10098 12382272 PMC4943216

[B53] Fritzsch B. Maklad A. Bruce L. L. Crapon De Caprona M. D. (2001). Development of the ear and of connections between the ear and the brain: is there a role for gravity? Adv. Space Res. 28, 595–600 10.1016/S0273-1177(01)00387-8 11803959

[B54] Fuller P. M. Jones T. A. Jones S. M. Fuller C. A. (2002). Neurovestibular modulation of circadian and homeostatic regulation: vestibulohypothalamic connection? Proc. Natl. Acad. Sci. U.S.A. 99, 15723–15728 10.1073/pnas.242251499 12434016 PMC137783

[B55] Gaboyard S. Sans A. Lehouelleur J. (2003). Differential impact of hypergravity on maturating innervation in vestibular epithelia during rat development. Brain Res. Dev. Brain Res. 143, 15–23 10.1016/S0165-3806(03)00069-5 12763577

[B56] Geisler H. C. Gramsbergen A. (1998). The EMG development of the longissimus and multifidus muscles after plugging the horizontal semicircular canals. J. Vestib. Res. 8, 399–409 10.1016/S0957-4271(97)00100-6 9842510

[B57] Geisler H. C. Westerga J. Gramsbergen A. (1996). The function of the long back muscles during postural development in the rat. Behav. Brain Res. 80, 211–215 10.1016/0166-4328(96)00024-1 8905145

[B58] Geleoc G. S. Holt J. R. (2003). Developmental acquisition of sensory transduction in hair cells of the mouse inner ear. Nat. Neurosci. 6, 1019–1020 10.1038/nn1120 12973354 PMC2669437

[B59] Gimenez Y Ribotta M. Sandillon F. Privat A. (1998). Influence of hypergravity on the development of monoaminergic systems in the rat spinal cord. Brain Res. Dev. Brain Res. 111, 147–157 10.1016/S0165-3806(98)00132-1 9838085

[B60] Gliddon C. M. Darlington C. L. Smith P. F. (2003a). Activation of the hypothalamic-pituitary-adrenal axis following vestibular deafferentation in pigmented guinea pig. Brain Res. 964, 306–310 10.1016/S0006-8993(02)04086-6 12576192

[B61] Gliddon C. M. Smith P. F. Darlington C. L. (2003b). Interaction between the hypothalamic-pituitary-adrenal axis and behavioural compensation following unilateral vestibular deafferentation. Acta Otolaryngol. 123, 1013–1021 10.1080/00016480310000520 14710901

[B62] Hara H. Sekitani T. Kido T. Endo S. Ikeda T. Takahashi M. (1995). Fine structures of utricle of developing chick embryo exposed to 2G gravity. Acta Otolaryngol. Suppl. 519, 257–261 10.3109/00016489509121918 7610881

[B63] Hensch T. K. (2004). Critical period regulation. Annu. Rev. Neurosci. 27, 549–579 10.1146/annurev.neuro.27.070203.144327 15217343

[B64] Hensch T. K. Fagiolini M. (2005). Excitatory-inhibitory balance and critical period plasticity in developing visual cortex. Prog. Brain Res. 147, 115–124 10.1016/S0079-6123(04)47009-5 15581701

[B65] Hensch T. K. Fagiolini M. Mataga N. Stryker M. P. Baekkeskov S. Kash S. F. (1998). Local GABA circuit control of experience-dependent plasticity in developing visual cortex. Science 282, 1504–1508 10.1126/science.282.5393.1504 9822384 PMC2851625

[B66] Heywood P. Pujol R. Hilding D. A. (1976). Development of the labyrinthine receptors in the guinea pig, cat and dog. Acta Otolaryngol. 82, 359–367 10.3109/00016487609120920 998205

[B67] Hoffman L. F. Ross M. D. Varelas J. Jones S. M. Jones T. A. (2006). Afferent synapses are present in utricular hair cells from otoconia-deficient mice. Hear. Res. 222, 35–42 10.1016/j.heares.2006.05.013 17023128

[B68] Horn E. R. (2004). “Critical periods” in vestibular development or adaptation of gravity sensory systems to altered gravitational conditions? Arch. Ital. Biol. 142, 155–174 15260375

[B69] Huang Z. J. Kirkwood A. Pizzorusso T. Porciatti V. Morales B. Bear M. F. (1999). BDNF regulates the maturation of inhibition and the critical period of plasticity in mouse visual cortex. Cell 98, 739–755 10.1016/S0092-8674(00)81509-3 10499792

[B70] Hubel D. H. Wiesel T. N. (1970). The period of susceptibility to the physiological effects of unilateral eye closure in kittens. J. Physiol. 206, 419–436 5498493 10.1113/jphysiol.1970.sp009022 PMC1348655

[B71] Inglis F. M. Zuckerman K. E. Kalb R. G. (2000). Experience-dependent development of spinal motor neurons. Neuron 26, 299–305 10.1016/S0896-6273(00)81164-2 10839350

[B72] Ito M. (2006). Cerebellar circuitry as a neuronal machine. Prog. Neurobiol. 78, 272–303 10.1016/j.pneurobio.2006.02.006 16759785

[B73] Jamon M. (2006). The early development of motor control in neonate rat. Comptes Rendus Palevol 5, 657–666 10.1016/j.crpv.2005.11.018

[B74] Jones S. M. Erway L. C. Johnson K. R. Yu H. Jones T. A. (2004). Gravity receptor function in mice with graded otoconial deficiencies. Hear. Res. 191, 34–40 10.1016/j.heares.2004.01.008 15109702

[B75] Jones S. M. Jones T. A. (2000). Ontogeny of vestibular compound action potentials in the domestic chicken. J. Assoc. Res. Otolaryngol. 1, 232–242 10.1007/s101620010026 11545229 PMC2504545

[B76] Kakizawa S. Yamasaki M. Watanabe M. Kano M. (2000). Critical period for activity-dependent synapse elimination in developing cerebellum. J. Neurosci. 20, 4954–4961 10864953 10.1523/JNEUROSCI.20-13-04954.2000 PMC6772278

[B77] Kawamata S. Igarashi Y. (1993). The fine structure of the developing otolithic organs of the rat. Acta Otolaryngol. Suppl. 504, 30–37 10.3109/00016489309128118 8470529

[B78] Keefe J. R. Alberts J. R. Krasnov I. B. Serova L. V. (1986). Developmental morphology of the eye, vestibular system and brain in 18-day fetal and newborn rats exposed *in utero* to null gravity during flight of Cosmos 1514. NASA Tech. Memo. 88223, 189–279

[B78a] Kerman I. A. Yates B. J. (1999). Patterning of somatosympathetic reflexes. Am. J. Physiol. 277, R716–R724 10484488 10.1152/ajpregu.1999.277.3.r716

[B79] Krasnov I. B. (1991). The otolith apparatus and cerebellar nodulus in rats developed under 2-G gravity. Physiologist 34, S206–S207 2047444

[B80] Krasnov I. B. Polyakov I. V. Ilyina-Kakueva E. I. Drobyshev V. I. (1992). Morphology and histochemistry of spinal cord and soleus muscle in rats grown under hypergravity. Physiologist 35, S216–S217 1589511

[B81] Ladd B. Nguon K. Sajdel-Sulkowska E. M. (2006). The effect of exposure to hypergravity on pregnant rat dams, pregnancy outcome and early neonatal development. Adv. Space Res. 38, 1100–1111 10.1016/j.asr.2005.07.049

[B82] Lim D. J. Stith J. A. Stockwell C. W. Oyama J. (1974). Observations on saccules of rats exposed to long-term hypergravity. Aerosp. Med. 45, 705–710 4837183

[B83] Liu D. Diorio J. Day J. C. Francis D. D. Meaney M. J. (2000). Maternal care, hippocampal synaptogenesis and cognitive development in rats. Nat. Neurosci. 3, 799–806 10.1038/77702 10903573

[B84] Lopez C. Blanke O. (2011). The thalamocortical vestibular system in animals and humans. Brain Res. Rev. 67, 119–146 10.1016/j.brainresrev.2010.12.002 21223979

[B84a] Lychakov D. V. Lavrova E. A. (1985). [Structure of the vestibular apparatus and ionic composition of the body of Xenopus laevis larvae as affected by weightlessness]. Kosm. Biol. Aviakosm. Med. 19, 48–52 3875754

[B85] Machado M. L. Kroichvili N. Freret T. Philoxène B. Lelong-Boulouard V. Denise P. (2012). Spatial and non-spatial performance in mutant mice devoid of otoliths. Neurosci. Lett. 522, 57–61 10.1016/j.neulet.2012.06.016 22705908

[B86] Maklad A. Fritzsch B. (2003a). Development of vestibular afferent projections into the hindbrain and their central targets. Brain Res. Bull. 60, 497–510 10.1016/S0361-9230(03)00054-6 12787869 PMC3901526

[B87] Maklad A. Fritzsch B. (2003b). Partial segregation of posterior crista and saccular fibers to the nodulus and uvula of the cerebellum in mice, and its development. Dev. Brain Res. 140, 223–236 10.1016/S0165-3806(02)00609-0 12586428

[B88] Malacinski G. M. Neff A. W. Alberts J. R. Souza K. A. (1989). Developmental biology in outer space: spaceflight provides the opportunity for new studies. Bioscience 39, 314–320 10.2307/1311114 11539116

[B89] Mandillo S. Del Signore A. Paggi P. Francia N. Santucci D. Mele A. (2003). Effects of acute and repeated daily exposure to hypergravity on spatial learning in mice. Neurosci. Lett. 336, 147–150 10.1016/S0304-3940(02)01282-X 12505614

[B90] Mbiene J. P. Favre D. Sans A. (1988). Early innervation and differentiation of hair cells in the vestibular epithelia of mouse embryos: SEM and TEM study. Anat. Embryol. (Berl.) 177, 331–340 10.1007/BF00315841 3354849

[B91] Meigal A. Y. (2013). Synergistic action of gravity and temperature on the motor system within the lifespan: a “Baby Astronaut” hypothesis. Med. Hypotheses 80, 275–283 10.1016/j.mehy.2012.12.004 23287049

[B92] Mitani K. Horii A. Kubo T. (2004). Impaired spatial learning after hypergravity exposure in rats. Cogn. Brain Res. 22, 94–100 10.1016/j.cogbrainres.2004.08.002 15561505

[B93] Moody S. A. Golden C. (2000). Developmental biology research in space: issues and directions in the era of the international space station: notes from the september 1999 meeting of the international space life sciences working group in woods hole, Massachusetts. Dev. Biol. 228, 1–5 10.1006/dbio.2000.9907 11087621

[B94] Moorman S. J. Cordova R. Davies S. A. (2002). A critical period for functional vestibular development in zebrafish. Dev. Dyn. 223, 285–291 10.1002/dvdy.10052 11836792

[B95] Muir G. D. (2000). Early ontogeny of locomotor behaviour: a comparison between altricial and precocial animals. Brain Res. Bull. 53, 719–726 10.1016/S0361-9230(00)00404-4 11165806

[B96] Murakami D. M. Erkman L. Hermanson O. Rosenfeld M. G. Fuller C. A. (2002). Evidence for vestibular regulation of autonomic functions in a mouse genetic model. Proc. Natl. Acad. Sci. U.S.A. 99, 17078–17082 10.1073/pnas.252652299 12466504 PMC139272

[B97] Nguon K. Ladd B. Baxter M. G. Sajdel-Sulkowska E. M. (2006a). Development of motor coordination and cerebellar structure in male and female rat neonates exposed to hypergravity. Adv. Space Res. 38, 1089–1099 10.1016/j.asr.2005.02.09517364014

[B98] Nguon K. Ladd B. Sajdel-Sulkowska E. M. (2006b). Exposure to altered gravity during specific developmental periods differentially affects growth, development, the cerebellum and motor functions in male and female rats. Adv. Space Res. 38, 1138–1147 10.1016/j.asr.2006.09.007 17364014 PMC1827157

[B99] Ornitz D. M. Bohne B. A. Thalmann I. Harding G. W. Thalmann R. (1998). Otoconial agenesis in tilted mutant mice. Hear. Res. 122, 60–70 10.1016/S0378-5955(98)00080-X 9714575

[B100] Pedrozo H. A. Schwartz Z. Luther M. Dean D. D. Boyan B. D. Wiederhold M. L. (1996). A mechanism of adaptation to hypergravity in the statocyst of *Aplysia californica*. Hear. Res. 102, 51–62 10.1016/S0378-5955(96)00147-5 8951450

[B101] Pellis V. C. Pellis S. M. Teitelbaum P. (1991). A descriptive analysis of the postnatal development of contact-righting in rats (*Rattus norvegicus*). Dev. Psychobiol. 24, 237–263 10.1002/dev.420240405

[B102] Peusner K. D. (2001). Development of the gravity sensing system. J. Neurosci. Res. 63, 103–108 10.1002/1097-4547(20010115)63:2<103::AID-JNR1001>3.3.CO;2-J 11169619

[B103] Phillips R. W. (2002). Gravity: it's the law. J. Gravit. Physiol. 9, P15–P16 14703665

[B104] Poo C. Isaacson J. S. (2007). An early critical period for long-term plasticity and structural modification of sensory synapses in olfactory cortex. J. Neurosci. 27, 7553–7558 10.1523/JNEUROSCI.1786-07.2007 17626216 PMC6672607

[B105] Porte Y. Morel J. L. (2011). Learning on jupiter, learning on the moon: the dark side of the G-force. Effects of gravity changes on neurovascular unit and modulation of learning and memory. Front. Behav. Neurosci. 6:64 10.3389/fnbeh.2012.00064 23015785 PMC3449275

[B106] Raymond J. Demêmes D. Blanc E. Dechesne C. J. (2003). Development of vestibular system in microgravity, in The Neurolab Spacelab Mission: Neuroscience Research in Space, eds Buckley J. C. Homick J. L. (Houston: National aeronautics and space Admnistration), 143–149

[B107] Rochefort C. Lefort J. M. Rondi-Reig L. (2013). The cerebellum: a new key structure in the navigation system. Front. Neural. Cir. 7:35 10.3389/fncir.2013.00035 23493515 PMC3595517

[B108] Ronca A. E. (2003). Mammalian development in space. Adv. Space Biol. Med. 9, 217–251 10.1016/S1569-2574(03)09009-9 14631635

[B109] Ronca A. E. Alberts J. R. (1997). Altered vestibular function in fetal and newborn rats gestated in space. J. Gravit. Physiol. 4, P63–P66 11540701

[B110] Ronca A. E. Alberts J. R. (2000). Effects of prenatal spaceflight on vestibular responses in neonatal rats. J. Appl. Physiol. 89, 2318–2324 11090585 10.1152/jappl.2000.89.6.2318

[B111] Ronca A. E. Fritzsch B. Alberts J. R. Bruce L. L. (2000). Effects of microgravity on vestibular development and function in rats: genetics and environment. Korean J. Biol. Sci. 4, 215–221 10.1080/12265071.2000.9647547 12760372

[B112] Ronca A. E. Fritzsch B. Bruce L. L. Alberts J. R. (2008). Orbital spaceflight during pregnancy shapes function of mammalian vestibular system. Behav. Neurosci. 122, 224–232 10.1037/0735-7044.122.1.224 18298265 PMC2610337

[B113] Ronca A. E. Lamkin C. A. Alberts J. R. (1993). Maternal contributions to sensory experience in the fetal and newborn rat (*Rattus norvegicus*). J. Comp. Psychol. 107, 61–74 10.1037/0735-7036.107.1.61 8444020

[B114] Ross M. D. Donovan K. Chee O. (1985). Otoconial morphology in space-flown rats. Physiologist 28, S219–S220 3834471

[B115] Roubertoux P. Semal C. Ragueneau S. (1985). Early development in mice: II. Sensory motor behavior and genetic analysis. Physiol. Behav. 35, 659–666 10.1016/0031-9384(85)90393-2 4080829

[B116] Sacher G. A. Staffeldt E. F. (1974). Relation of gestation time to brain weight for placental mammals: implications for the theory of vertebrate growth. Am. Nat. 108, 595–615 10.1086/282938

[B117] Sajdel-Sulkowska E. M. (2008). Brain development, environment and sex: what can we learn from studying graviperception, gravitransduction and the gravireaction of the developing CNS to altered gravity? Cerebellum 7, 223–239 10.1007/s12311-008-0001-8 18418693

[B118] Sajdel-Sulkowska E. M. Li G. H. Ronca A. E. Baer L. A. Sulkowski G. M. Koibuchi N. (2001). Effects of hypergravity exposure on the developing central nervous system: possible involvement of thyroid hormone. Exp. Biol. Med. (Maywood) 226, 790–798 11520946 10.1177/153537020222600812

[B119] Sajdel-Sulkowska E. M. Nguon K. Sulkowski Z. L. Rosen G. D. Baxter M. G. (2005). Purkinje cell loss accompanies motor impairment in rats developing at altered gravity. Neuroreport 16, 2037–2040 10.1097/00001756-200512190-00014 16317350

[B120] Santucci D. Francia N. Trincia V. Chiarotti F. Aloe L. Alleva E. (2009). A mouse model of neurobehavioural response to altered gravity conditions: an ontogenetical study. Behav. Brain Res. 197, 109–118 10.1016/j.bbr.2008.08.008 18775454

[B121] Santucci D. Kawano F. Ohira T. Terada M. Nakai N. Francia N. (2012). Evaluation of gene, protein and neurotrophin expression in the brain of mice exposed to space environment for 91 days. PLoS ONE 7:e40112 10.1371/journal.pone.0040112 22808101 PMC3392276

[B122] Sarkar P. Sarkar S. Ramesh V. Hayes B. E. Thomas R. L. Wilson B. L. (2006). Proteomic analysis of mice hippocampus in simulated microgravity environment. J. Proteome Res. 5, 548–553 10.1021/pr050274r 16512669 PMC2748658

[B123] Savel'ev S. V. Serova L. V. Besova N. V. Nosovskii A. M. (1998). [Effect of weightlessness on rats endocrine system development]. Aviakosm. Ekolog. Med. 32, 31–36 9661773

[B124] Sekulic S. Lukac D. Drabsin M. Suknjaja V. Kekovic G. Grbic G. (2009). The righting reflex from a supine to a prone position in the guinea pig fetus. Gen. Physiol. Biophys. 28 Spec No, 284–288 19893112

[B125] Sekulic S. R. Lukac D. D. Naumovic N. M. (2005). The fetus cannot exercise like an astronaut: gravity loading is necessary for the physiological development during second half of pregnancy. Med. Hypotheses 64, 221–228 10.1016/j.mehy.2004.08.012 15607544

[B126] Serova L. V. (1991). Hypergravity and development of mammals. Physiologist 34, S135–S136 2047411

[B127] Serova L. V. Denisova L. A. Pustynnikova A. M. (1985). Comparative analysis of hypo - and hypergravity effects on prenatal development of mammals. Physiologist 28, S5–S8 3834483

[B128] Shiroyama T. Kayahara T. Yasui Y. Nomura J. Nakano K. (1999). Projections of the vestibular nuclei to the thalamus in the rat: a *Phaseolus vulgaris* leucoagglutinin study. J. Comp. Neurol. 407, 318–332 10.1002/(SICI)1096-9861(19990510)407:3<3C318::AID-CNE2>3E3.3.CO;2-8 10320214

[B129] Simons D. J. Land P. W. (1987). Early experience of tactile stimulation influences organization of somatic sensory cortex. Nature 326, 694–697 10.1038/326694a0 3561512

[B130] Smith M. Yuan Wang X. Wolgemuth D. J. Murashov A. K. (2003). Development of the mouse vestibular system in the absence of gravity perception. Brain Res. Dev. Brain Res. 140, 133–135 10.1016/S0165-3806(02)00591-6 12524184

[B131] Smith P. F. Geddes L. H. Baek J. H. Darlington C. L. Zheng Y. (2010). Modulation of memory by vestibular lesions and galvanic vestibular stimulation. Front. Neurol. 1:141 10.3389/fneur.2010.00141 21173897 PMC2995955

[B132] Sobin A. Anniko M. (1983). Embryonic development of the specific vestibular hair cell pathology in a strain of the waltzing guinea pig. Acta Otolaryngol. 96, 397–405 10.3109/00016488309132725 6605653

[B133] Sondag H. N. De Jong H. A. Oosterveld W. J. (1997). Altered behaviour in hamsters conceived and born in hypergravity. Brain Res. Bull. 43, 289–294 10.1016/S0361-9230(97)00008-7 9227839

[B134] Sondag H. N. De Jong H. A. Van Marle J. Oosterveld W. J. (1995). Effects of sustained acceleration on the morphological properties of otoconia in hamsters. Acta Otolaryngol. 115, 227–230 10.3109/00016489509139297 7610810

[B135] Sondag H. N. De Jong H. A. Van Marle J. Willekens B. Oosterveld W. J. (1996). Otoconial alterations after embryonic development in hypergravity. Brain Res. Bull. 40, 353–356 discussion: 357. 10.1016/0361-9230(96)00127-X 8886358

[B136] Stackman R. W. Taube J. S. (1997). Firing properties of head direction cells in the rat anterior thalamic nucleus: dependence on vestibular input. J. Neurosci. 17, 4349–4358 9151751 10.1523/JNEUROSCI.17-11-04349.1997 PMC1489676

[B137] Straka H. (2010). Ontogenetic rules and constraints of vestibulo-ocular reflex development. Curr. Opin. Neurobiol. 20, 689–695 10.1016/j.conb.2010.06.003 20637600

[B138] Sun X.-Q. Xu Z.-P. Zhang S. Cao X.-S. Liu T.-S. (2009). Simulated weightlessness aggravates hypergravity-induced impairment of learning and memory and neuronal apoptosis in rats. Behav. Brain Res. 199, 197–202 10.1016/j.bbr.2008.11.035 19100783

[B139] Taube J. S. (2007). The head direction signal: origins and sensory-motor integration. Annu. Rev. Neurosci. 30, 181–207 10.1146/annurev.neuro.29.051605.112854 17341158

[B140] Tees R. C. (1967). Effects of early auditory restriction in the rat on adult pattern discrimination. J. Comp. Physiol. Psychol. 63, 389–393 10.1037/h0024619 6064381

[B141] Temple M. D. Kosik K. S. Steward O. (2002). Spatial learning and memory is preserved in rats after early development in a microgravity environment. Neurobiol. Learn. Mem. 78, 199–216 10.1006/nlme.2001.4049 12431413

[B142] Trinel D. Picquet F. Bastide B. Canu M.-H. (2013). Dendritic spine remodeling induced by hindlimb unloading in adult rat sensorimotor cortex. Behav. Brain Res. 249, 1–7 10.1016/j.bbr.2013.04.015 23608484

[B143] Van Kan C. M. De Vries J. I. P. LüChinger A. B. Mulder E. J. H. Taverne M. A. M. (2009). Ontogeny of fetal movements in the guinea pig. Physiol. Behav. 98, 338–344 10.1016/j.physbeh.2009.06.011 19560478

[B144] Ventre-Dominey J. Nighoghossian N. Denise P. (2005). Interaction between cortical control of vestibular function and spatial representation in man. Ann. N.Y. Acad. Sci. 1039, 494–497 10.1196/annals.1325.052 15827007

[B145] Vetter D. E. Mann J. R. Wangemann P. Liu J. Mclaughlin K. J. Lesage F. (1996). Inner ear defects induced by null mutation of the isk gene. Neuron 17, 1251–1264 10.1016/S0896-6273(00)80255-X 8982171

[B146] Vidal P. P. Degallaix L. Josset P. Gasc J. P. Cullen K. E. (2004). Postural and locomotor control in normal and vestibularly deficient mice. J. Physiol. 559, 625–638 10.1113/jphysiol.2004.063883 15243133 PMC1665125

[B147] Vinay L. Ben-Mabrouk F. Brocard F. Clarac F. Jean-Xavier C. Pearlstein E. (2005). Perinatal development of the motor systems involved in postural control. Neural. Plast. 12, 131–139 discussion: 263–172. 10.1155/NP.2005.131 16097481 PMC2565459

[B148] Vinay L. Brocard F. Pflieger J. F. Simeoni-Alias J. Clarac F. (2000). Perinatal development of lumbar motoneurons and their inputs in the rat. Brain Res. Bull. 53, 635–647 10.1016/S0361-9230(00)00397-X 11165799

[B149] Wade C. E. (2005). Responses across the gravity continuum: hypergravity to microgravity. Adv. Space Biol. Med. 10, 225–245 10.1016/S1569-2574(05)10009-4 16101110

[B150] Walton K. (1998). Postnatal development under conditions of simulated weightlessness and space flight. Brain Res. Brain Res. Rev. 28, 25–34 10.1016/S0165-0173(98)00023-X 9795115

[B151] Walton K. D. Benavides L. Singh N. Hatoum N. (2005a). Long-term effects of microgravity on the swimming behaviour of young rats. J. Physiol. 565, 609–626 10.1113/jphysiol.2004.074393 15760948 PMC1464537

[B152] Walton K. D. Harding S. Anschel D. Harris Y. T. Llinas R. (2005b). The effects of microgravity on the development of surface righting in rats. J. Physiol. 565, 593–608 10.1113/jphysiol.2004.074385 15774538 PMC1464515

[B153] Walton K. D. Kalb R. Defelipe J. Garcia-Segura M. Hillman D. Llinas R. (2003). Motor system development depends on experience, in The Neurolab Spacelab Mission: Neuroscience Research in Space, eds Buckley J. C. Homick J. L. (Houston: National aeronautics and space Admnistration), 95–104

[B154] Walton K. D. Lieberman D. Llinas A. Begin M. Llinas R. R. (1992). Identification of a critical period for motor development in neonatal rats. Neuroscience 51, 763–767 10.1016/0306-4522(92)90517-6 1488121

[B155] Wang V. Y. Zoghbi H. Y. (2001). Genetic regulation of cerebellar development. Nat. Rev. Neurosci. 2, 484–491 10.1038/35081558 11433373

[B156] Wiederhold M. L. Gao W. Harrison J. L. Parker K. A. (2003a). Early development of gravity sensing organs in microgravity, in The Neurolab Spacelab Mission: Neuroscience Research in Space, eds Buckley J. C. Homick J. L. (Houston: National aeronautics and space Admnistration), 123–132

[B157] Wiederhold M. L. Harrison J. L. Gao W. (2003b). A critical period for gravitational effects on otolith formation. J. Vestib. Res. 13, 205–214 15096664

[B158] Wiederhold M. L. Gao W. Y. Harrison J. L. Hejl R. (1997). Development of gravity-sensing organs in altered gravity. Gravit. Space Biol. Bull. 10, 91–96 11540125

[B158a] Wong A. M. Desantis M. (1997). Rat gestation during space flight: outcomes for dams and their offspring born after return to Earth. Integr. Physiol. Behav. Sci. 32, 322–342 10.1007/BF02688630 9502520

[B159] Wood C. (1970). Weightlessness: its implications for the human fetus. J. Obstet. Gynaecol. Br. Commonw. 77, 333–336 10.1111/j.1471-0528.1970.tb03528.x 5427295

[B160] Wubbels R. J. De Jong H. A. (2000). Vestibular-induced behaviour of rats born and raised in hypergravity. Brain Res. Bull. 52, 349–356 10.1016/S0361-9230(00)00279-3 10922513

[B161] Wubbels R. J. Van Marle J. Sondag H. N. De Jong H. A. (2002). Effects of hypergravity on the morphological properties of the vestibular sensory epithelium. II. Life-long exposure of rats including embryogenesis. Brain Res. Bull. 58, 575–580 10.1016/S0361-9230(02)00828-6 12372561

[B162] Yates B. J. Holmes M. J. Jian B. J. (2003). Plastic changes in processing of graviceptive signals during spaceflight potentially contribute to postflight orthostatic intolerance. J. Vestib. Res. 13, 395–404 15096680

[B163] Yoder R. M. Taube J. S. (2009). Head direction cell activity in mice: robust directional signal depends on intact otolith organs. J. Neurosci. 29, 1061–1076 10.1523/JNEUROSCI.1679-08.2009 19176815 PMC2768409

[B164] Zheng J. L. Gao W. Q. (1997). Analysis of rat vestibular hair cell development and regeneration using calretinin as an early marker. J. Neurosci. 17, 8270–8282 9334402 10.1523/JNEUROSCI.17-21-08270.1997 PMC6573764

